# Dynamics of Mechanical Signal Transmission through Prestressed Stress Fibers

**DOI:** 10.1371/journal.pone.0035343

**Published:** 2012-04-13

**Authors:** Yongyun Hwang, Abdul I. Barakat

**Affiliations:** 1 Hydrodynamics Laboratory (LadHyX), Department of Mechanics, École Polytechnique, CNRS UMR7646, Palaiseau, France; 2 Mechanical & Aerospace Engineering, University of California Davis, Davis, California, United States of America; Dalhousie University, Canada

## Abstract

Transmission of mechanical stimuli through the actin cytoskeleton has been proposed as a mechanism for rapid long-distance mechanotransduction in cells; however, a quantitative understanding of the dynamics of this transmission and the physical factors governing it remains lacking. Two key features of the actin cytoskeleton are its viscoelastic nature and the presence of prestress due to actomyosin motor activity. We develop a model of mechanical signal transmission through prestressed viscoelastic actin stress fibers that directly connect the cell surface to the nucleus. The analysis considers both temporally stationary and oscillatory mechanical signals and accounts for cytosolic drag on the stress fibers. To elucidate the physical parameters that govern mechanical signal transmission, we initially focus on the highly simplified case of a single stress fiber. The results demonstrate that the dynamics of mechanical signal transmission depend on whether the applied force leads to transverse or axial motion of the stress fiber. For transverse motion, mechanical signal transmission is dominated by prestress while fiber elasticity has a negligible effect. Conversely, signal transmission for axial motion is mediated uniquely by elasticity due to the absence of a prestress restoring force. Mechanical signal transmission is significantly delayed by stress fiber material viscosity, while cytosolic damping becomes important only for longer stress fibers. Only transverse motion yields the rapid and long-distance mechanical signal transmission dynamics observed experimentally. For simple networks of stress fibers, mechanical signals are transmitted rapidly to the nucleus when the fibers are oriented largely orthogonal to the applied force, whereas the presence of fibers parallel to the applied force slows down mechanical signal transmission significantly. The present results suggest that cytoskeletal prestress mediates rapid mechanical signal transmission and allows temporally oscillatory signals in the physiological frequency range to travel a long distance without significant decay due to material viscosity and/or cytosolic drag.

## Introduction

Mechanical forces regulate cellular growth, differentiation, motility, and apoptosis through pathways that remain incompletely understood. The mechanisms governing cellular mechanotransduction, the process by which cells sense mechanical forces and transduce these forces into biochemical signals, are currently under intense investigation. Mechanochemical conversion in cells often initiates at or near the cell membrane and is mediated by specific surface molecules [1], [2] including mechanosensitive ion channels [3]–[6], integrins [7], the cellular glycocalyx [8], cell-cell adhesion complexes [9], and G protein-coupled receptors [10]. Activation of protein kinases [11], [12] and other membrane-associated signaling pathways rapidly ensues. Ultimately, mechanical stimulation activates transcription factors, leading to force-dependent changes in gene expression and protein synthesis.

The cytoskeleton is intricately involved in cellular mechanotransduction. Mechanical forces induce rapid cytoskeletal deformation [13], regulate cytoskeletal organization [14], and activate acto-myosin motors [15] as well as protein kinases bound to cytoskeletal elements (such as Src) [16], [17]. Of particular relevance to the present study, actin stress fibers have been reported to directly transmit mechanical stimuli applied to integrins on the cell surface to the nucleus [18], thereby potentially regulating nuclear ion channels [19], [20], transcription/splicing factors [21], and ultimately gene expression. A key feature of mechanical stimulus transmission through stress fibers is that it allows much faster long-distance mechanotransduction than is possible via diffusion- and reaction-limited membrane receptor-driven signaling cascades. For instance, mechanical stimuli transmitted via the cytoskeleton have been reported to travel a distance of 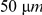 in less than 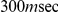, while chemical signaling cascades require more than 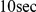 to travel the same distance [17], [21].

Actin stress fibers in cells are in a state of ‘prestress’ (pre-existing isometric tension) [22], [23]. Experiments have shown that disrupting the actin cytoskeleton or dissipating cytoskeletal prestress inhibits rapid long-distance cellular mechanotransduction [17], [24]–[28]. This suggests that cytoskeletal prestress plays an important role in long-distance mechanical signal transmission. It has recently been conjectured that rapid mechanical signal transmission occurs via elastic waves in stress fibers [17], [21], a seemingly plausible mechanism in light of the fact that elastic waves in stress fibers are estimated to travel at a velocity of 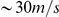. However, stress fibers are not elastic structures but rather viscoelastic as has been clearly demonstrated in recent experiments of fiber retraction following laser severing [23]. Importantly, that study suggested that the time scale for viscoelastic retraction is on the order of a few seconds, orders of magnitude larger than the microsecond time scale derived from the elastic wave argument. Therefore, the same experiments that show that stress fibers bear prestress also demonstrate the strong viscoelastic nature of actin stress fibers, casting doubt on the notion that rapid and long-distance mechanical signal transmission by stress fibers occurs via elastic wave propagation.

Although experimental findings indicate that prestress in actin stress fibers is critical for rapid long-distance mechanical signal transmission [17], [26], [27], a physical understanding of this process is lacking. The present study aims to elucidate the physical factors that govern the dynamics of mechanical signal transmission through prestressed actin stress fibers using a relevant mathematical model. To extract fundamental information on the physical factors that determine the time scale for stress transmission, we begin by considering the highly simplified case of a single stress fiber. The underlying assumption is that mechanical signals are transmitted via force-induced deformation of the stress fiber, and the effects of stress fiber prestress and viscoelastic behavior as well as cytosolic drag on this deformation are computed. The governing equations are solved numerically, and the results are supplemented with dimensional analysis to provide insight into the dominant physical processes involved in mechanical signal transmission. Whenever possible, the results are also discussed vis-a-vis available experimental data. The paper concludes by extending the analysis to simple stress fiber networks in order to investigate the effects of cytoskeletal connections on the dynamics of mechanical signal transmission.

## Methods

### Model of a prestressed cytoskeletal filament

**Figure 1:**
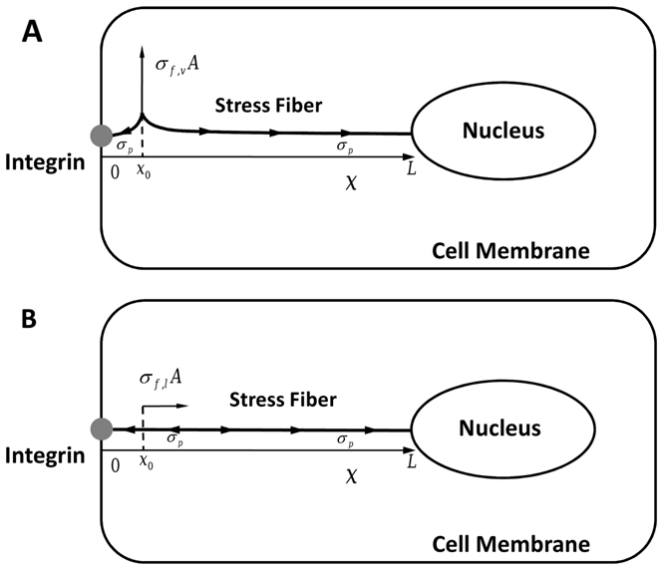
Schematic diagram of the present model for 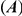 transverse and 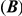 axial motion. The integrin is positioned at 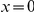 and the nuclear edge at 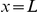. An actin stress fiber of length 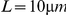 directly links the integrin to the nucleus. A prestress 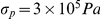 is assumed to be uniformly distributed throughout the stress fiber. The transverse and axial forces are applied at 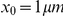.

We consider the highly simplified configuration where a viscoelastic cytoskeletal filament directly links an integrin on the cell surface to the nucleus as depicted in Fig. 1. We assume that the cytoskeletal filament is an actin stress fiber because stress fibers have been implicated in mechanical force transmission [17], [24]–[28] (we will briefly discuss microtubules and intermediate filaments later). Stress fibers generate prestress mainly due to actomyosin motor activity [23]; thus, we assume that prestress 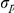 is uniformly distributed along the stress fiber length. The length of the stress fiber (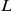) is chosen as 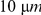, a representative length scale in many eukaryotic cells. This length is considerably shorter than the persistence length (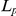) of a typical stress fiber (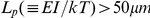 [29], where 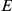 is the elastic modulus, 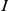 the second moment of inertia, 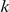 the Boltzman constant and 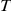 the absolute temperature; 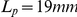 using the parameters in the present study). The long persistence length allows us to neglect stochastic motion caused by thermal effects and therefore to consider a purely deterministic description of stress fiber motion.

We first consider the transverse motion of the stress fiber driven by a forcing with stress amplitude 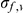 applied at 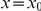 as depicted in Fig. 1*A*. In this case, prestress acts as a restoring force in the transverse direction in a manner similar to tension in a string [30]. Therefore, we combine the prestress-associated restoring force with the Euler-Bernoulli beam equation. The stress fiber is assumed to be a viscoelastic material whose constitutive relation is given by the Kelvin-Voigt model as verified by recent experimental observations [23]. Cytosolic drag on the moving stress fiber is derived assuming Stokes flow and is implemented as an external damping force. The resulting equation of stress fiber transverse motion, assuming small-scale deformation, is written as:
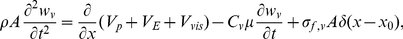
(1a)where
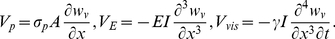
(1b)Here, 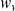 is the transverse (or vertical) displacement of the stress fiber, 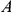 the cross-sectional area of the fiber, 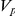 the restoring force by prestress 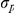, 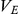 the restoring force by flexural rigidity 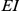, 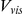 the internal damping force by the flexural material viscosity 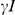, 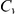 the cytosolic resistance coefficient for transverse motion, 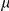 the viscosity of the cytosol, and 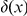 the Dirac delta function. At the integrin, we impose a stress-free (and hence force-free) boundary condition so that:
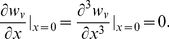
(1c)At the nucleus, we consider a ‘pinched’ boundary condition whereby:
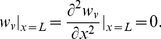
(1d)Here, zero displacement is imposed because the nucleus is considerably more rigid than cytoskeletal elements. The second boundary condition at the nucleus, which denotes a moment-free boundary, is chosen to allow force transmission to the nucleus.

Axial motion is also considered and is schematically depicted in Fig. 1*B*. Forcing is applied at 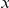 = 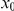 with a stress amplitude 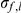. In this case, contrary to transverse motion, prestress does not act as a restoring force, and it changes the stress fiber's elasticity and material viscosity only when the axial displacement is sufficiently large to result in nonlinear viscoelastic behavior [31], a situation which is not considered here. Therefore, maintaining the Kelvin-Voigt model for the viscoelastic constitutive relation, the equation for axial motion is given as:
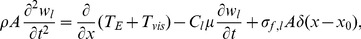
(2a)where
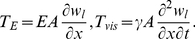
(2b)Here, 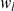 is the axial (or longitudinal) displacement of the stress fiber, 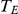 the restoring force by the elastic modulus 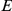, 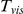 the internal damping force by the material viscosity 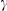, and 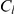 the cytosolic resistance coefficient for axial motion. Stress-free and zero displacement boundary conditions are applied to the integrin and the nucleus, respectively so that:
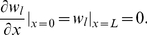
(2c)


**Figure 2:**
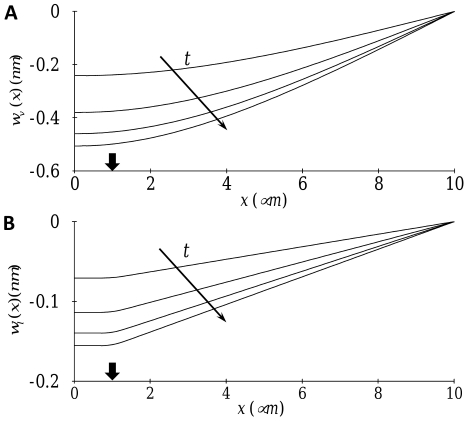
Temporal evolution of stress fiber displacement: (A) transverse motion 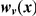 at 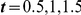 and 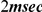; (B) axial motion 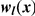 at 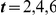 and 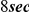. Arrows indicate the positions of stress application (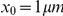).

It should be noted that the stress-free condition imposed at the integrin for both transverse and axial motion allows movement of the stress fiber when the forcing is applied near the integrin (see also Fig. 2), which roughly mimics experiments where force is directly applied using magnetic or optical tweezers to a microbead bound to integrins on the cell surface. For this reason, the forcing location is chosen near the integrin (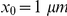). We also note that the present formulation is not restricted to an integrin-nucleus link but is equally applicable to the situation where a stress fiber directly links integrins on the cell surface to other relatively rigid cellular structures such as focal adhesion sites, cell-cell adhesion proteins, etc. In that case, the boundary condition at any of these other sites would be similar to that presented here for the nucleus. Therefore, the governing system considered here can in principle also be used to extract information on the dynamics of mechanical signal transmission via stress fibers to other intracellular sites.

### Model parameter values

Table 1 summarizes the geometric and mechanical charcteristics of the stress fiber considered in the present study. The stress fiber is assumed to be a circular cylinder composed of a homogeneous mixture of actin filaments and cross-linking proteins. The stress fiber radius is set to 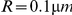 [23], [31], which leads to the cross sectional area 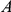 and the second moment of area 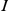 values provided in the table. The density of the stress fiber 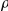 is assumed to be that of water (also that of an actin filament [29]); this value has previously been used to estimate the speed of an elastic wave in a stress fiber [17]. Prestress within the stress fiber is computed (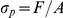) from a recent experiment [31] where the pre-existing tension of isolated stress fibers from smooth muscle cells was measured as 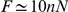. The elastic modulus is also obtained from the same experiment [31]. While the elastic modulus of a stress fiber is generally a nonlinear function of axial strain, it remains virtually constant in the tension range 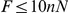 [31]. Thus, because force amplitudes used in mechanotransduction studies are typically very small (for instance, a stress of less than 20 Pa is sufficient for Src activation in smooth muscle cells [17]), we assume a constant value of the elastic modulus as given in Table 1. The material viscosity of the stress fiber 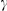 is assumed to be constant and is obtained from a recent report of the time constant associated with the retraction of viscoelastic stress fibers after laser severing [23]: 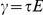, where 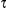 is the time constant. For cytosolic drag, the reference value of the cytosolic viscosity 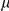 is assumed to be that of water. The cytosolic transverse and longitudinal resistance coefficients 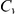 and 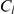 are approximated using a Stokes flow assumption as detailed in Materials S1.

**Table 1:** Parameters values for the present model.

	Reference value	Test range	Source
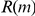	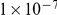	-	[23], [31]
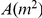	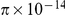	-	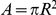
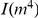	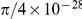	-	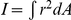
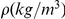	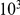	-	[17]
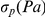	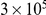	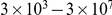	[31]
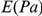	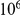	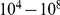	[31], [32], [43]
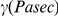	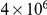	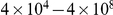	[23]
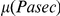	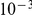	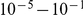	-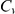	1	-	Materials S1
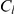	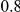	-	Materials S1

The individual contributions of prestress, elasticity, material viscosity, and cytoplasmic drag are systematically studied by examining a large range of their values. This parametric study is also important since the nominal values chosen for the reference case may have non-negligible deviation. For instance, a recent measurement showed that the elastic modulus is 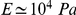 [32], which is two orders of magnitude smaller than the reference value in Table 1. Also, physiological prestress can be an order of magnitude smaller than the reference value since the radius of a stress fiber varies in the range of 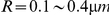 [23], [32].

### Numerical methods

Equations (1) and (2) are numerically solved using the finite difference method [33]. The axial direction is uniformly discretized using a second-order central difference scheme with 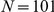 grid points. The Dirac delta function in the forcing term is approximated using a Gaussian with sufficiently narrow width: 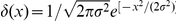 with 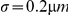. Time-integration is conducted semi-implicitly with second-order accuracy [33]: the stress transport terms related to prestress and elasticity are advanced using a third-order low-storage Runge-Kutta method, and the material viscosity and cytosolic drag terms are integrated using the second-order Crank-Nicolson method. The code is implemented in Fortran 90 and is validated with a resolution test for the reference parameters. More specifically, the computational results (e.g. time constant of the strain at the nucleus) with the present resolution show approximately 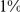 difference from results with 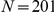. All computations in this study were carried out on an Intel Xeon CPU E5345 operating Linux.

## Results and Discussion

To examine mechanical signal transmission through the stress fiber, we consider both constant and purely oscillatory time-periodic forcing applied to an initially stationary stress fiber: i.e. an initial condition of zero displacement and zero velocity. Constant forcing is applied with a very short time ramp to avoid numerical errors associated with abrupt switching: 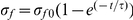 where 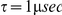. For the same reason, oscillatory forcing is considered as a sinusoidal function in time: 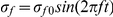 where 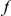 is the forcing frequency. For both constant and oscillatory forcing, the amplitude of the forcing is chosen as 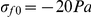 (the minus sign implies downward and outward pulling for transverse and axial motion, respectively), which is sufficiently large to elicit a bilogical cellular response (e.g. Src activation) [17]. Because (1) and (2) are linear systems, the forcing amplitude does not change the conclusions presented in this paper.

It has been suggested that force-induced biological responses in cells may be attributable to mechanical deformation of proteins that bind signaling molecules [17]. Therefore, we assume that mechanical signal transmission to the nucleus occurs via stress fiber deformation and define the relevant mechanical signal transmitted to the nucleus as the deformation-related stress at the nucleus. For transverse motion, the deformation-related stress has both prestress and elasticity contributions and takes the following form:
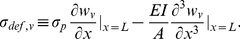
(3a)For axial motion, on the other hand, only elasticity contributes to deformation, which leads to the following expression for deformation-related stress at the nucleus:
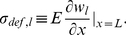
(3b)


### Constant forcing

**Figure 3:**
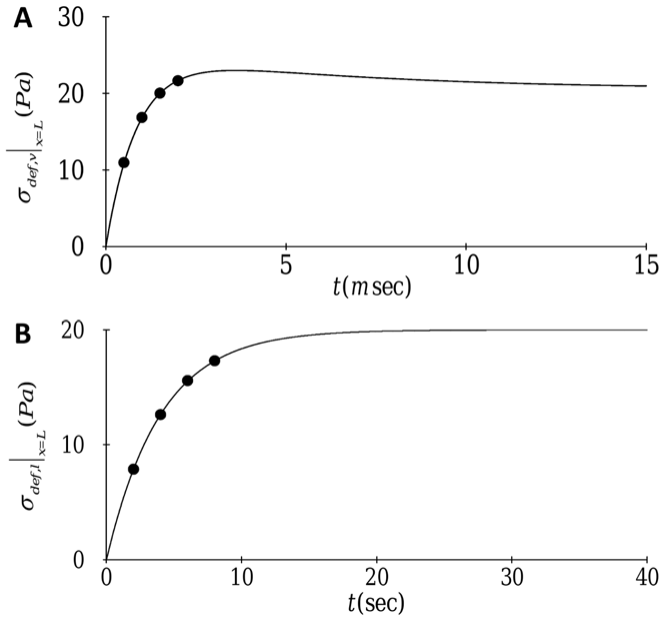
Temporal evolution of the deformation-related stress at the nucleus (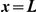): (A) transverse motion; (B) axial motion. Dots indicate the time corresponding to the displacement snapshots in Fig. 2.

We first consider mechanical signal transmission driven by constant forcing. The temporal evolution of transverse and axial displacements for the reference parameter values defined in Table 1 is depicted in Fig. 2. The stress fiber displacements increase progressively with time for both the transverse and axial directions; however, the transverse motion exhibits a much faster response. This can be seen more clearly in Fig. 3 which illustrates the temporal evolution of deformation-related stress at the nucleus. For transverse motion, the deformation-related stress at the nucleus attains the applied stress (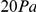) within a few milliseconds, whereas axial stress requires several seconds for transmission of the applied stress to the nucleus. It should be noted that both of these time scales are considerably longer than the few microseconds predicted by the conjecture of elastic wave stress transmission where material viscosity and cytosolic damping are neglected [17], [21]. These results suggest that material viscosity and/or cytosolic damping play crucial roles in delaying mechanical signal transmission. A more detailed discussion of this notion is provided later.

**Figure 4:**
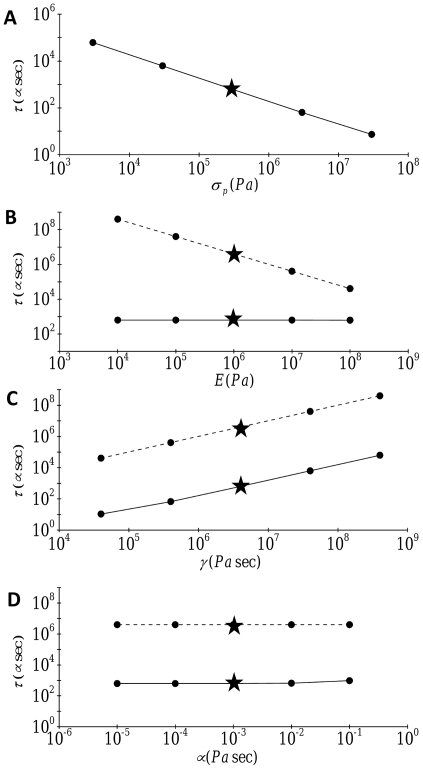
Effect of (A) prestress, (B) elasticity, (C) material viscosity, and (D) cytosolic viscosity on the time constant 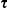 for the deformation-related stress evolution at the nucleus (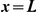):––––, transverse motion; width 3.429 pt height 0.5 pt width 3.429 pt height 0.5 pt width 3.429 pt height 0.5 pt width 3.429 pt height 0.5 pt, axial motion. Stars denote the reference values.

We investigated the individual roles of physical parameters including prestress, stress fiber viscoelastic properties, and cytosolic viscosity by conducting a parametric study on the time constant characterizing mechanical signal transmission to the nucleus (defined as the time required for deformation-related stress at the nucleus to reach 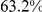 of the applied stress). The range of the parameters studied is summarized in Table 1. In this parametric study, each parameter of interest was varied individually while all other parameters were maintained at their reference values. Fig. 4 illustrates the dependence of the time constant on prestress, stress fiber elasticity, stress fiber material viscosity, and cytosolic viscosity for both transverse and axial motion. For transverse motion, an increase in stress fiber prestress significantly reduces the time constant for mechanical signal transmission to the nucleus (Fig. 4*A*). The effect of stress fiber elasticity is found to be negligible for transverse motion; however, for axial motion, an increase in elasticity results in a significant reduction in the time constant because elasticity constitutes the only mechanism for stress propulsion in this case (Fig. 4*B*). These results suggest that cytoskeleton-mediated mechanical signal transmission from the cell surface to the nucleus occurs through different mechanisms depending on the direction of the force: a transverse mechanical stimulus is mainly propelled by prestress while axial stimulus transmission is mediated by cytoskeletal elasticity. These different propelling processes are significantly delayed by the material viscosity which leads to internal damping; thus, the time constant for both transverse and axial mechanical signal transmission increases with an increase in material viscosity (Fig. 4*C*). Cytosolic drag, on the other hand, plays a negligible role in delaying mechanical signal transmission in both the transverse and axial directions as evidenced by the fact that the time constant for mechanical signal transmission is virtually independent of cytosolic viscosity (Fig. 4*D*). These findings indicate that for the configuration considered here, material viscosity is the dominant dragging mechanism of mechanical signal transmission.

### Oscillatory forcing

**Figure 5:**
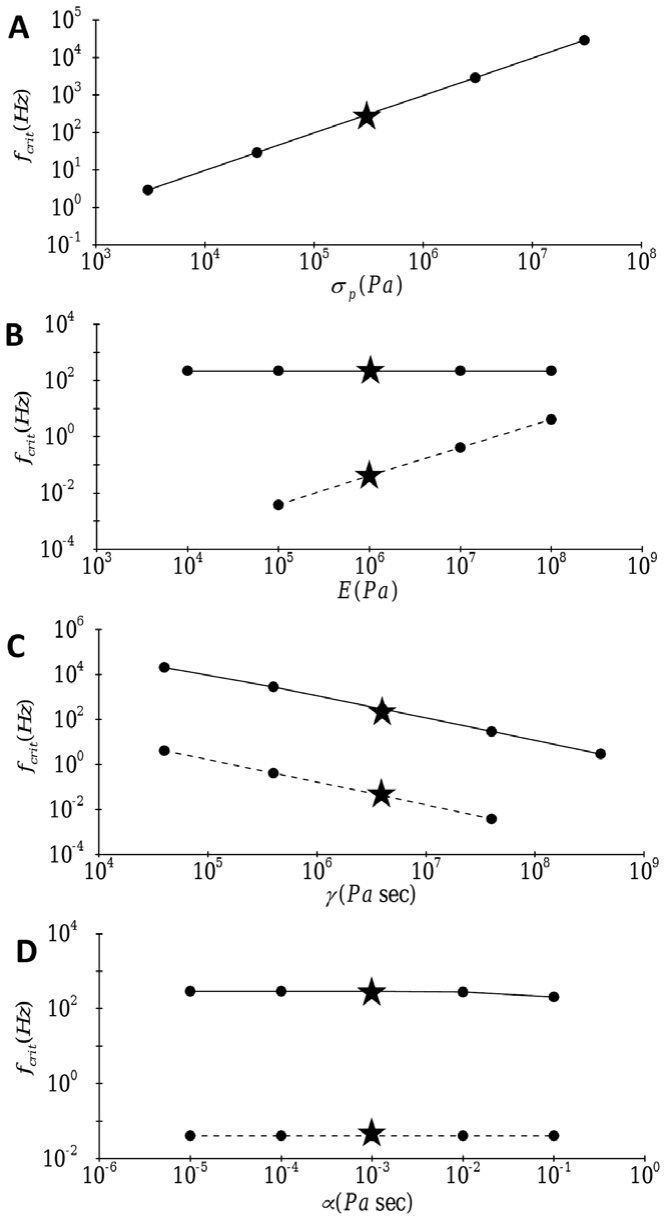
Time trace of oscillatory forcing (- - - -) and the resulting deformation-related stress (––––) at the nucleus: 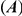 transverse motion with 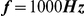; 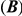 axial motion with 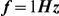.

The results above were derived using a constant forcing function. There is ample evidence that cells respond differently to oscillatory forcing than they do to constant forcing [34]–[36]; therefore, we have also studied the transmission of a time-periodic mechanical stimulus. The specific waveform we consider is a sinusoidally oscillating stress with a zero mean and an amplitude of 20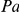. Fig. 5 illustrates representative time traces for the deformation-related stress at the nucleus for oscillatory forcing applied in both the transverse and axial directions. For both directions, the deformation-related stress, as in the case of constant forcing, exhibits an initial transient before eventually settling to a time-periodic steady state response with a saturation amplitude and a frequency matching that of the applied forcing frequency (1000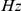 for transverse forcing and 1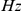 for axial forcing).

**Figure 6:**
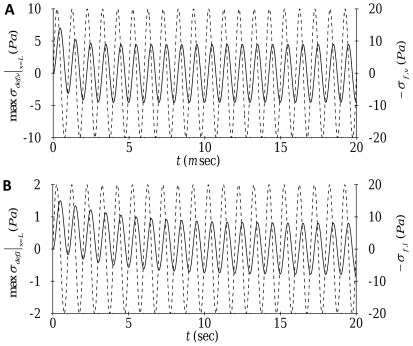
Effect of the forcing frequency on the amplitude of the deformation-related stress at the nucleus (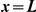):––––, transverse motion; - - - -, axial motion.

As expected, the saturation amplitude of the deformation-related stress at the nucleus is strongly dependent on the forcing frequency as illustrated in Fig. 6. For both transverse and axial motion, low frequency forcing is transmitted to the nucleus without a decay in amplitude. However, for a sufficiently large forcing frequency, the deformation-related stress at the nucleus progressively decays with an increase in frequency, although transverse motion also exhibits very slight amplification of the applied forcing around 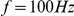. These results suggest that the governing equations (1) and (2) are strongly damped linear systems; thus, individual stress fibers behave as low-pass filters of mechanical forcing. It is noteworthy that transverse motion exhibits a much broader filter width than axial motion: the filter width for transverse motion extends to 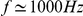 whereas the filter width for axial motion extends only to 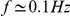.

**Figure 7:**
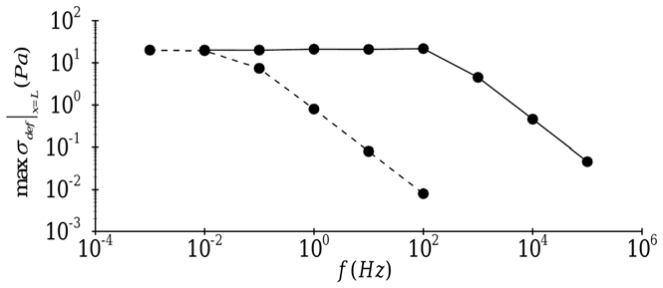
Effect of (A) prestress, (B) elasticity, (C) material viscosity, and (D) cytosolic viscosity on the critical frequency 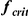 of the deformation-related stress evolution at the nucleus (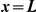):––––, stress in the transverse direction (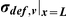); - - - -, stress in the longitudinal direction (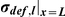). Stars denote the reference values.

We studied the individual roles of physical parameters including prestress, stress fiber viscoelastic properties, and cytosolic viscosity on the frequency response by computing the critical frequency below which full signal transmission is achieved (i.e. stress fiber filter width). This critical frequency is defined as the frequency at which the amplitude of deformation-related stress at the nucleus equals to 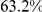 of the amplitude of the applied forcing. Because a wide range of forcing frequencies is considered, the critical frequency was obtained using a third-order spline interpolation of the computed data for peak stress at the nucleus as a function of each of the physical parameters of interest plotted on log-log axes. Fig. 7 depicts the dependence of the critical frequency on prestress, elasticity and material viscosity of the stress fiber, and cytosolic viscosity. For transverse motion, prestress significantly increases the critical frequency (Fig. 7*A*) whereas, similar to constant forcing, the effect of elasticity is negligible (Fig. 7*B*). For axial motion, on the other hand, an increase in elasticity leads to a significant increase in the critical frequency (Fig. 7*B*). These results suggest that for oscillatory forcing, the filter widths for transverse and axial motion are determined by different mechanisms. For both transverse and axial motion, an increase in stress fiber material viscosity significantly reduces the critical frequency (Fig. 7*C*), whereas cytosolic damping plays a negligible role (Fig. 7*D*). These findings indicate that material viscosity plays a dominant role as a system damper in mechanical signal transmission, similar to the case of constant forcing.

### Prestress mediates rapid long-distance mechanical signal transmission

The results of the previous sections suggest that the primary propellant of cytoskeleton-mediated mechanical signal transmission is prestress for force applied in the transverse direction and cytoskeletal elasticity for force applied in the axial direction. To further understand the mechanisms of mechanical signal transmission, we use Eqs. (1) and (2) to derive expressions for the energy exchange rates for both transverse and axial motion as detailed in Materials S1. For transverse motion, the equation for energy exchange rate is written as:
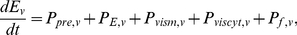
(4a)where 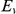 is the kinetic energy for transverse motion, and 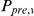, 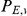, 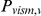, 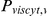 and 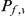 are respectively the work rates by prestress, elasticity, material viscosity, cytosolic drag, and the driving forcing. Similarly, the equation for axial motion is written as:
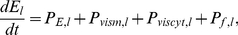
(4b)where 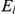 is the kinetic energy for axial motion, and 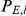, 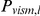, 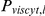 and 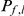 are respectively the work rates by elasticity, material viscosity, cytosolic drag, and the driving forcing.

**Figure 8:**
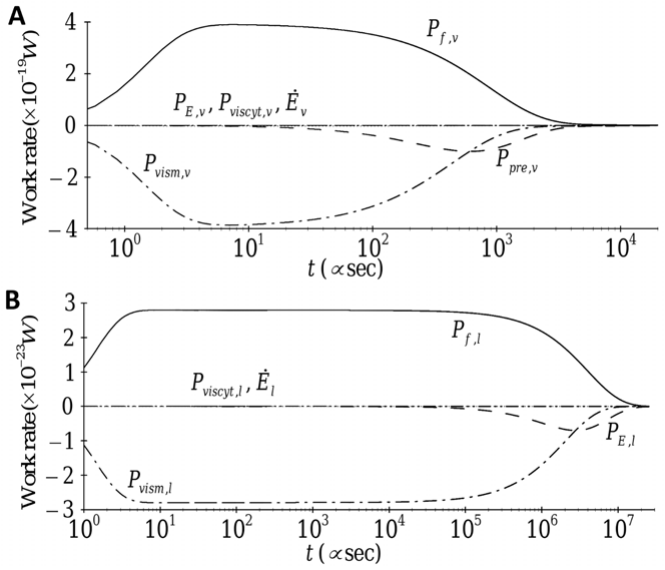
Time evolution of the individual work-rate contribution of each term for the 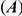 transverse and 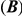 longitudinal components of the applied force.

Fig. 8 depicts time traces of the work rate associated with each term for both transverse and axial motion for the case of constant forcing. At steady state, all work-rate terms drop off to zero (because each term is multiplied by the stress fiber velocity). Immediately after forcing is applied, material viscosity plays the dominant role in reducing the velocity of the stress fiber for both transverse and axial motion. With the decrease in velocity, the material viscosity terms 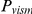 drop off to zero faster than the forcing terms 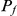 because 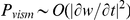 whereas 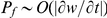. Following decay of the material viscosity term, the driving force is borne by the prestress term 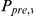 in the case of transverse motion and by the elasticity term 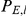 for axial motion, resulting in deformation of the stress fiber. The contributions of the remaining terms (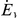, 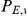 and 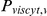 for transverse motion and 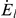 and 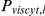 for axial motion) are negligible.

An important difference between transverse and axial motion in Fig. 8 is the time at which the terms related to deformation (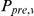 for transverse motion and 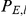 for axial motion) begin to play important roles. This time is 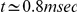 for transverse motion and 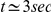 for axial motion. These time scales can also be obtained using dimensional analysis by balancing the dominant terms for each motion. Thus, for transverse motion, setting 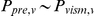, yields:
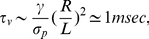
(5a)where 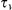 is the time scale of deformation in the transverse direction. Similarly, for axial motion, the time scale of deformation 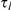 is obtained from the balance of 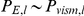 and yields:
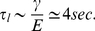
(5b)These two time scales are consistent with the results of Fig. 4 which had shown that 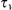 is proportional to 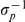 and 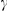 but is virtually independent of the elastic modulus and cytosolic viscosity and that 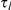 is proportional to 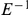 and 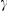 but is independent of the cytosolic viscosity.

As already alluded to, the time scales for transverse and axial motion 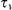 and 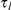 are both several orders of magnitude larger than the time scale obtained from the elastic wave conjecture which neglects material and cytosolic damping [17], [21]. This suggests that the damping terms significantly delay mechanical signal transmission. Taking this delay into account leads to the observation that only the time scale for transverse motion 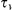 is consistent with the experimental observation that mechanical deformations are transmitted a distance of 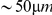 in less than 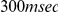 [17]. On the other hand, the time scale for axial motion 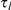 deviates significantly from this experimental observation. It should be noted that 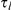 is consistent with the experimental result demonstrating that viscoelastic retraction of a stress fiber in the axial direction occurs with a time scale of 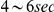 [23]. Taken together, the present results suggest that mechanical stimulus transmission through transverse stress fiber motion is likely the only pathway for very rapid mechanotransduction and that it is mediated primarily by prestress.

The time scales in (5a) and (5b) also provide insight into the results for oscillatory forcing. Since the time scale is an inherent feature of a given system, its inverse provides the characteristic frequency of the system. Thus, the characteristic frequencies for transverse and axial motion 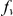 and 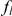 are as follows:
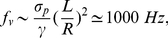
(6a)

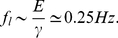
(6b)This dimensional prediction is consistent with the critical frequency values in Figs. 6 and 7.

Physiological time-periodic mechanical stimuli are often characterized by frequencies in the range 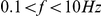 (e.g. cardiac and respiratory frequencies). The frequency scaling in (6a) and (6b) suggests that for mechanical signal transmission via an actin stress fiber, only transverse stress fiber motion allows time-periodic mechanical signal in the physiological frequency range to be transmitted a long distance within a cell. In contrast, a physiologically relevant time-periodic mechanical signal transmitted via axial stress fiber motion would not propagate deeply into a cell because material viscosity rapidly dampens the mechanical signal. Importantly, the transverse motion critical frequency above which internal viscosity significantly dampens mechanical signal transmission is directly proportional to the prestress in the stress fiber as was illustrated in Fig. 7. This suggests that when prestress is reduced, this critical frequency would be significantly lowered so that mechanical signals in the physiological frequency range would be rapidly damped, preventing long distance mechanical signal transmission. This prediction is in qualitative agreement with the experimental finding that dissipation of stress fiber prestress by pharmacological agents (e.g. caldesmon) inhibits long-distance mechanical stimulus transmission in smooth muscle cells [24], [26]–[28]. Despite this encouraging qualitative agreement, the complex physiology of the cytoskeleton and its intracellular connections complicate efforts at more quantitative predictions by the present model. For instance, while experimental studies have suggested that low levels of caldesmon which significantly inhibit transmission of a 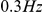 oscillatory signal dissipate approximately 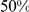 of the existing cytoskeletal prestress [27], the present model predicts that a 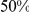 reduction in prestress leads to a critical frequency of 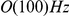, implying that a time-periodic mechanical signal with a frequency of 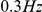 would be transmitted through a stress fiber.

An additional implication of our numerical and dimensional analysis for steady and harmonic forcing is that the nature of mechanical signal transmission in an actin stress fiber is fundamentally different for transverse motion than for axial motion. For transverse motion, prestress plays an essential role as a stiffness, while the effect of material stiffness, i.e. flexural rigidity (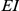), is negligible. This notion is confirmed by the following dimensional analysis:
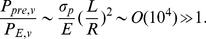
(7)Prestress also renders mechanical signal transmission through transverse motion dependent on stress fiber aspect ratio (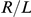) (see (5a) and (6a)), and this indeed is an essential reason why transverse motion provides rapid long-distance mechanical signal transmission. Importantly, the time scale for transverse motion in (5a) is inversely proportional to the square of the length, suggesting that a longer stress fiber would more rapidly transmit mechanical signal. For axial motion, mechanical signal transmission is dependent only on mechanical properties (see (5b) and (6b)); thus, the geometry of the stress fiber does not influence the speed of mechanical signal transmission.

### Material and cytosolic damping

**Figure 9:**
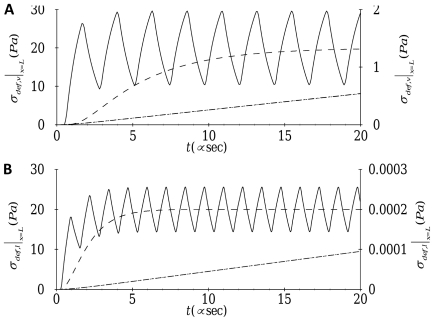
Time trace of the deformation-related stress at the nucleus (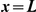). (A) Stress in the transverse direction (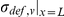) with only the reference prestress (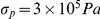, 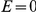): ––––, without cytosolic or material viscosity (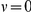, 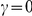); - - - -, with only cytosolic viscosity (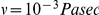, 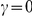); –-–-–-–, with only material viscosity (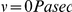, 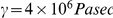); –-–-–-, with both cytosolic and material viscosities (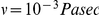, 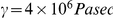). (B) Stress in the axial direction (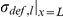) with the reference elasticity (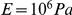):––––, without cytosolic or material viscosity (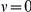, 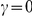); - - - -, with only cytosolic viscosity (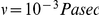, 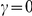); –-–-–, with only material viscosity (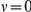, 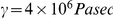); –-–-–, with both cytosolic and material viscosities (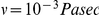, 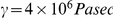). Note that the dash-dot and dash-double dot traces overlap and that the time traces that include material viscosity are amplified using the axes in the right in order to better visualize their very small values.

In the present analysis, both material viscosity and cytosolic drag act as damping factors that slow down mechanical signal transmission through a stress fiber. For the reference parameters, we have shown that material viscosity is the dominant factor in slowing down mechanical signal transmission. To further delineate the relative contributions of material and cytosolic damping, Fig. 9 provides time traces of deformation-related stress of transverse and axial motion at the nucleus for the following four scenarios: 1) no damping, 2) cytosolic damping only, 3) material damping only, and 4) both material and cytosolic damping. Without any damping, prestress and elasticity act as restoring forces for transverse and axial motion, respectively. Therefore, both transverse and axial motion exhibit elastic oscillations of the deformation-related stress due to the generation and reflection of elastic waves (solid lines in Fig. 9). Indeed, the behavior of axial motion is identical to that conjectured in [17], [21]: the elastic wave travels with a velocity 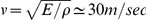; thus, the nucleus begins to deform at 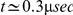 (Fig. 9B). When only cytosolic damping is considered, the elastic oscillations in stress for both transverse and axial motion completely disappear (dashed lines in Fig. 9); however, the time constant for both cases is on the order of several microseconds, indicating that cytosolic damping, while effective at damping stress oscillations, plays only a partial role in delaying mechanical signal transmission. Material viscous damping plays a much more prominent role in delaying mechanical signal transmission as evidenced by the fact that both transverse and axial motion with only material viscous damping exhibit dynamics that are virtually identical to those of the reference cases where all damping terms are included (overlapping dashed-dot and dashed-double-dot lines in Fig. 9).

Dimensional analysis of material and cytosolic damping provides further insight. The ratio of cytosolic damping to material damping for transverse motion is given as:
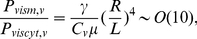
(8a)while the same ratio for axial motion is:
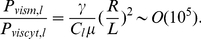
(8b)Here, it is interesting to note that the ratio for transverse motion is only 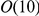 whereas that for axial motion is 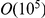. This suggests that the influence of cytosolic damping is much larger for transverse motion than for axial motion. Moreover, depending on cell size, 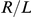 may attain values of 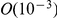 in which case the ratio for transverse motion in Eq. (8a) would become 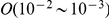. Thus, the contribution of cytosolic damping to delaying transverse mechanical stimuli transmission is expected to be considerably larger for stress fibers that are longer than those considered in the present study.

### Microtubules and intermediate filaments

Thus far, we have only discussed mechanical signal transmission through a single actin stress fiber; however, the cytoskeleton also contains microtubules, intermediate filaments, and a variety of linker proteins. Mechanical signal transmission through the cytoskeleton is likely to be significantly affected by the mechanical properties of these other cytoskeletal elements as well as by the detailed networking of these various elements.

Microtubules are thought to primarily bear compressive loads [37], [38]. Since the persistence length of a microtubule is of order 1 mm [39], it would appear that setting prestress to an appropriate negative value and applying appropriate boundary conditions in Eq. (1) (e.g. 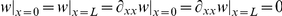) may provide physical insight into the dynamics of a weakly compressed microtubule. For example, if we consider a Fourier-Laplace mode for this case with zero material and cytosolic damping (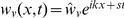 where 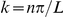 for 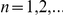), the following threshold of compressional force 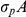 for the Euler-buckling instability is obtained with the critical wavenumber 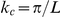:
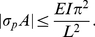
(9)The flexural rigidity of a microtubule has been reported as 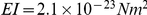 [40], which yields a critical force 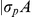 for microtubule buckling on the order of 1 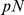. However, this probably does not accurately represent microtubule behavior in cells. Indeed, the buckling wavelength of a microtubule in vivo has been found to be considerably shorter than 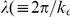)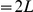 predicted by Eq. (19) due to the coupling of microtubules to surrounding cytoskeleton [38]. Importantly, this coupling appears to allow microtubules in vivo to bear loads up to 100 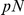, two orders of magnitude larger than those predicted by the Euler buckling analysis. Moreover, microtubules in cells are often highly bent [38], suggesting that studying mechanical signal transmission through microtubules with the present model, which only considers small deformations of a straight filament, may not be appropriate.

Similar to stress fibers, intermediate filaments are tensile cytoskeletal elements that bear large tensile forces [18]. Therefore, one may expect mechanical signal transmission through intermediate filaments to be similar to that through stress fibers. However, the persistence length of an intermediate filament is only on the order of 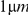 [39], [41]. Therefore, a deterministic description of mechanical signal transmission through an intermediate filament with a length of order 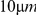 is not adequate due to its strong stochastic nature. Although intermediate filaments may form bundles in vivo [42], the mechanical properties of these bundles (most notably prestress and material viscosity) have not been well established, which limits the application of the present model to the case of intermediate filaments.

### Simple stress fiber networks

In cells, cytoskeletal elements are very commonly linked together via a variety of linker proteins. There is also mounting evidence that cytoskeletal coupling to the nucleus occurs through specialized linker proteins such as nesprins [21]. Naturally, the nature of all these links will affect the deformations of cytoskeletal elements in response to an applied force and hence will influence the dynamics of mechanical signal transmission considered here. For a single stress fiber, the present results indicate that transverse motion allows rapid long-distance mechanical signal transmission. In the case of a network of linked stress fibers, one can envision particular linking configurations that preserve rapid long-distance mechanical signal transmission whose dynamics are consistent with those observed in experiments [17], [24]–[28].

**Figure 10:**
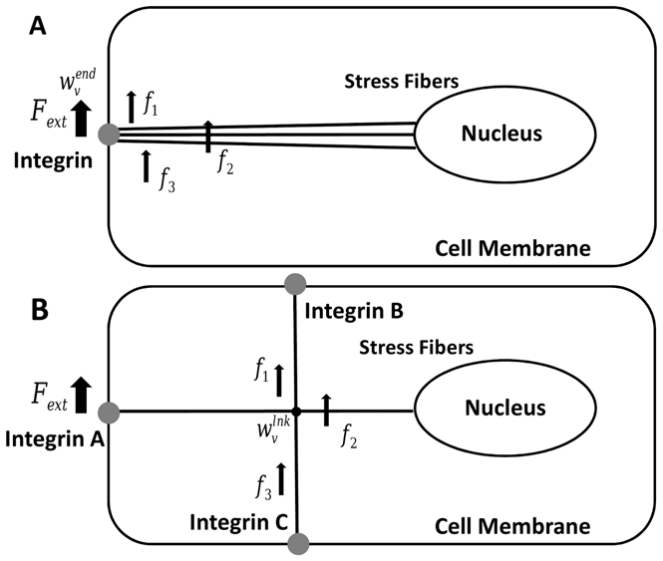
Examples of simple stress fiber network topologies. 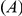
 Three stress fibers aligned nearly parallel to one another and linked together at their ends, and 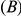 two stress fibers aligned perpendicular to one another with one of them constrained to move only in the axial direction.

A representative example is the case where several stress fibers are aligned virtually parallel to one another and linked together at their ends, as shown in Fig. 10 (*A*). In this case, a force applied in the transverse direction at the integrin (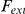) will lead to primarily transverse motion in each of the stress fibers; thus, the time scale for transmission of deformation-related stress would be virtually identical to that in 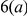:
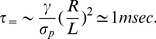
(10)It should be noted that this rapid mechanical signal transmission is essentially due to the absence of axial motion in the linked stress fiber network which, had it occurred, would have significantly slowed down mechanical signal transmission through each of the stress fibers.

In contrast, other links that allow the axial motion of one or more stress fibers to interfere with the transverse motion of other stress fibers would be expected to prevent rapid mechanical signal transmission. A representative situation is depicted in Fig. 10 (*B*), where two stress fibers are rigidly aligned perpendicular to one another with one of them allowed to move only in the axial direction. If a force is applied in the transverse direction at the integrin, this force will be transmitted to the node linking the two fibers. This transmitted force will then be redistributed to each stress fiber under the constraint of equal deformation and velocity for both fibers at the linker node. Considering the dominant terms involved in force transmission through each stress fiber, the force balance at the linker node (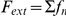 in Fig. 10*B*) leads to the following dimensional relation:
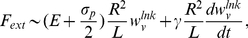
(11)where 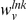 is the vertical deformation of the linker node. Note that, in this relation, the material damping force for transverse motion of the stress fiber aligned horizontally is neglected because it is much smaller than the material damping force related to axial motion of the stress fiber aligned vertically. Since the deformation at the linker node is proportional to the deformation-related stress transmitted to the nucleus, the time scale for the nucleus to ‘feel’ the mechanical deformation is given by:
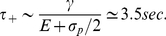
(12)This time scale is of the same order as the one in 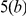, implying that the network topology in Fig. 10 (*B*) significantly slows down mechanical signal transmission to the nucleus despite the transverse motion of some of the actin stress fibers.

The predictions of the present model for stress fiber networks can be tested experimentally. Culturing cells on patterned surfaces organizes stress fibers in the direction of the pattern [25]. Our model predicts that mechanical signals applied to the cell surface in a direction orthogonal to the substrate pattern would get transmitted to the nucleus much more rapidly than stresses applied in the direction of the pattern. The present findings also have interesting and potentially important implications for normal physiology and pathology. Stress fiber orientation is a major determinant of cell shape, and cell shape often regulates cell function. A vivid example is the case of the arterial endothelium. In vivo, arterial regions prone to the development of atherosclerosis are often associated with cuboidal (nearly round) endothelial cells whose stress fibers are randomly oriented, whereas arterial zones with elongated endothelial cells and highly aligned stress fibers remain largely spared of the disease. Thus, the stress fiber topologies in Figs. 10 (*A*) and 10 (*B*) can be thought of as representative of elongated and cuboidal endothelial cells, respectively. It would be particularly interesting to establish if the differences in mechanical signal transmission dynamics between these two topologies predicted by our present model relate in any way to the observed functional differences between cuboidal and elongated endothelial cells. Establishing such a relationship promises to significantly enhance our understanding of the role of mechanical forces in the development and progression of atherosclerosis.

## Supporting Information

Materials S1 (DOC)
