# Correction: Dynamics of Mechanical Signal Transmission through Prestressed Stress Fibers

**DOI:** 10.1371/annotation/5eb90275-b1a3-4743-a75a-dc3e5d97c4fe

**Published:** 2012-07-10

**Authors:** Yongyun Hwang, Abdul I. Barakat

The images for Figures 5, 6, and 7 were incorrectly switched. The image that appears as Figure 6 should be Figure 5, and the image that appears as Figure 5 should be Figure 7. The image that appears as Figure 7 should be Figure 6. The figure legends appear in the correct order. 

