# Supplementary material for: Dynamics of Mechanical Signal Transmission through Prestressed Stress Fibers
Source: PLoS One. 2012 Apr 13;7(4):e35343. doi: 10.1371/journal.pone.0035343 (PMC3325979; doi:10.1371/journal.pone.0035343)
Supplement: Materials S1 — (DOC) [file pone.0035343.s001.doc]

Supplementary Materials

S1. Model Parameter Values

The geometric and mechanical characteristics of the stress fiber considered in the present study are summarized in Table 1. The stress fiber is assumed to be a circular cylinder composed of a homogeneous mixture of actin filaments and cross-linking proteins. The stress fiber radius is set to *R* = 0*.*1 *μm* (1, 2), which leads to the cross sectional area *A* and the second moment of area *I* values provided in the table. The density of the stress fiber *ρ* is assumed to be that of water (also that of an actin filament (3)); this value has previously been used to estimate the speed of an elastic wave in a stress fiber (4). Prestress within the stress fiber is computed (*σ_p_* = *F/A*) from a recent experiment (2) where the pre-existing tension of isolated stress fibers from smooth muscle cells was measured as *F ≈* 10 *nN*. The elastic modulus is also obtained from the same experiment (2). While the elastic modulus of a stress fiber is generally a nonlinear function of axial strain, it remains virtually constant in the tension range *F ≤*10 *nN* (2). Thus, because force amplitudes used in mechanotransduction studies are typically very small (for instance, a stress of less than 20 Pa is sufficient for Src activation in smooth muscle cells (4)). we assume a constant value of the elastic modulus as given in Table 1. The material viscosity of the stress fiber is assumed to be constant and is obtained from a recent report of the time constant associated with the retraction of viscoelastic stress fibers after laser severing (1):  *γ*= *τE*, where *τ* is the time constant. For cytosolic drag, the reference value of the cytosolic viscosity *μ* is assumed to be that of water. The cytosolic transverse and longitudinal resistance coefficients *Cv* and *Cl* are approximated using a Stokes flow assumption as detailed in section S3 (see below).

S2. Numerical Methods

Equations (1) and (2) are numerically solved using the finite difference method (5, 6). The axial direction is uniformly discretized using a second-order central difference with N = 101 grid points. The Dirac delta function in the forcing term is approximated using a Gaussian with sufficiently narrow width: $\delta(x)=1/\sqrt{2\pi\sigma^{2}}e^{\left[-x^{2}/2\sigma^{2}\right]}$ with *σ* = 0.2 *μm*. Time-integration is conducted semi-implicitly with second-order accuracy (6): stress transport terms related to prestress and elasticity are advanced using the third-order low-storage Runge-Kutta method, and material viscous and the cytosolic drag terms are integrated using the second-order Crank-Nicolson method. The code is implemented in Fortran 90 and is validated with a resolution test for the reference parameters. More specifically, the computational results (e.g. time constant of the strain at the nucleus) with the present resolution show approximately 1% difference from results with N = 201. All computations in this study were carried out on an Intel Xeon CPU E5345 operating Linux.

S3. Determination of resistance coefficients *C_v_* and *C_l_*

Here, we describe the detailed procedure used for obtaining the resistance coefficients for cytosolic damping. For transverse motion, *C_v_* is obtained by assuming that the hydrodynamic drag on a stress fiber is equivalent to that of an infinitely long cylinder subject to the free-stream velocity *V*. The free-stream velocity *V* is estimated as 10^-3^ *m/sec* since the maximum transverse displacement and the minimum time scale are assumed to be *O*(1 *nm*) and *O*(1 *μsec*), respectively. This estimation, and assuming the cytosol to have the dynamic viscosity of water, gives Re ~ *O*(10^-7^), with a corresponding drag coefficient of *C_v_* ≈ 1 following (7). Therefore, we set *C_v_* = 1 in all simulations. For axial motion, *C_l_* is computed by assuming that the axial hydrodynamic drag can be approximated as that on a circular cylinder moving axially in an outer circular tube. The radius of the outer tube is set as 1 ~ 10 *μ*m, and this results in *C_l_* ≈ 0.3 ~ 0.9 under Stokes flow conditions. For convenience, we choose *C_l_* = 0.8 in all simulations.

S4. Computation of energy exchange rate

Here, we show the details of terms in the work-rate equations (4a) and (4b). We multiply Eqs. (1a) and (2a) by *v_v_ ≡ ∂w_v_ / ∂t and v_l_ ≡ ∂w_l_ / ∂t,* respectively and integrate over x. Thus, Eq. (1) for transverse motion becomes:

(S1)

and Eq. (2) for axial motion becomes:

(S2)

References

1. Kumar, S., I. Z. Maxwell, A. Heisterkamp, T. R. Polte, T. Lele, M. Salanga, E. Mazur, and D. E. Ingber, 2006. Viscoelastic retraction of single living stress fibers and its impact on cell shape, cytoskeletal organization and extracellular matrix mechanics. *Biophys. J.* 90:3762-73.
2. Deguchi, S., T. Ohashi, and M. Sat, 2006. Tensile properties of single stress fibers isolated from cultured vascular smooth muscle cells. *Journal* *of Biomechanics* 39:2603-10.
3. Boal, D., 2002. Mechanics of the Cell. Cambridge University Press, Cambridge, first edition.
4. Na, S., O. Collin, F. Chowdhury, B. Tay, M. Ouyang, Y. Wang, and N. Wang, 2008. Rapid signal transduction in living cells is a unique feature of mechanotransduction. *Proc. Natl Acad. Sci. USA* 105:6626-31.
5. Moin, P., 2001. Fundamentals of engineering numerical analysis. Cambridge University Press, Cambridge, first edition.
6. Bewley, T. R., 2008. Numerical Renaissance: Simulation, Optimization and Control. Renaissance Press, San diego.
7. Zdravkovich, M. M., 1997. Flow around circular cylinders. Oxford Science Publication, Oxford, 1st edition.
